# Supplementary material for: Molecular Cytogenomic Characterization of the Murine Breast Cancer Cell Lines C-127I, EMT6/P and TA3 Hauschka
Source: Int J Mol Sci. 2020 Jul 1;21(13):4716. doi: 10.3390/ijms21134716 (PMC7369978; doi:10.3390/ijms21134716)
Supplement: Supplementary file 1 [file ijms-21-04716-s001.pdf]

Supplementary

Table 1. Regions of gains and loss of copy numbers, as well as breakpoints of balanced rearrangements, observed in the three different BC cell lines studied here and the corresponding homologue regions in humans are listed as cytoband and genomic position acc. to GRCh37(hg19).

**BC cell line C-127I**			
**region**	**gain**	**homologue region in human**	
		cytoband	position (GRCh37/hg19)
4qA1-qC4	1x	8q12.1-q12.3	8:56650304-62695565
		8q21.3-q22.1	8:87057363-97246782
		8q12.3	8:63094926-64018516
		6q14.3-q16.2	6:87793887-100245013
		9p21.2-p13.1	9:27325073-38472099
		9q22.33-q33.2	9:100037894-123488942
		9q21.31-q21.32	9:82993521-85697078
		9q21.32	9:85856924-86154717
5qF - qter	1x	12q24.31-q24.33	12:121577100-132336561
		7p11.2	7:56019352-56184138
		7q11.21-q11.22	7:66808098-72045725
		7q11.23	7:72536306-76149827
		7q22.1	7:99552841-102191754
		7p22.3-p22.1	7:169204-6771649
		7q21.3-q22.1	7:97598308-99229367
		13q12.13-q13.2	13:26784894-34260463
10qC1 - qD3	2x	19p13.3	19:281181-4173052
		12q23.3	12:104359309-108176937
		22q12.3	22:32783299-33472414
		12q13.2-q23.3	12:55351591-104351507
11qA1 – qB3	1x	5q33.1-q33.2	5:150381711-154330989
		1q42.13	1:227919753-228703212
		17p11.2	17:16917258-21343117
		17p12-p11.2	17:15731601-16472951
		22q12.1-q12.2	22:29251511-32022116
		7p13-p11.2	7:43906144-55317931
		2p16.2-p14	2:53882943-68694726
		5q35.1-q35.2	5:172736725-173663599
		5q33.2-q35.1	5:154331837-171932313
		5q35.3	5:177531363-180585244
		5q23.3-q31.1	5:130484032-134063627
		5q33.1-q33.2	5:150381711-154330989
11qB4 - qtr	1x (in 40%)	17p12-p11.2	17:15731601-16472951
		17p13.3-p12	17:2-15625804
		17q11.1-q11.2	17:25525650-28853901
		17q11.2-q12	17:29058377-36200511
		17q21.32-q23.2	17:45560334-60326198
		17q12-q21.31	17:36351926-43638822
		17q21.31-q21.32	17:43706746-45150591
		17q21.32	17:45188646-45518436
		17q23.2-q24.1	17:60483588-62760387
		17q24.1-q24.2	17:62990972-66110690
		17q24.2-q25.3	17:66224207-81175056

13qA1-qB1	1x (in 30%)	1q42.3-q43	1:235330060-240084659
		7p14.2-p13	7:36524506-43605930
		6p22.3-p22.1	6:20065223-28502803
		6p25.3-p23	6:181261-15099150
		6p23-p22.3	6:15104709-20060798
		9q22.1-q22.32	9:91031851-97067712
		5q35.2-q35.3	5:173750964-177039611
		5q31.1-q31.2	5:134073478-137090938
15qA1 - qtr	2x	5p15.31-p12	5:8927745-42888975
		8q22.1-q24.3	8:97446632-146158346
		22q12.3-q13.33	22:35962951-51222438
		12p11.1	12:33476533-34210697
		12q12-q13.2	12:38607141-55072925
17qA1 – qter	1x (in 40%) 2x(in 40%)	6q25.2-q25.3	6:155053083-160101646
		6q27	6:167120855-167552070
		6q25.3-q27	6:160103032-166797236
		6q27	6:167859539-170893754
		5q15-q21.1	5:96202316-98405239
		16p13.3	16:222880-3208490
		5q35.1	5:171946752-172722349
		6p21.32-p21.2	6:33359177-39058058
		21q22.3	21:43490502-45122943
		19p13.12	19:15270296-15808207
		19p13.2	19:8366687-8811037
		6p22.1-p21.32	6:29322703-33297218
		6p21.2-p12.3	6:39266498-49681826
		3p25.1-p24.3	3:16307846-20231899
		2q12.2-q12.3	2:107383985-108798215
		19p13.3	19:4229082-6862967
		5q21.1-q22.1	5:102759315-110063021
		18p11.32-p11.22	18:2534401-9972541
		2p23.2-p16.3	2:29033520-51699597
		2p16.3-p16.2	2:51709987-53282184
		18p11.32	18:861722-2534400
19qA1-qter	1x(in 36%) 2x(in 60%)	11q12.1-q13.3	11:57844834-68709722
		9q21.11-q21.31	9:69086307-82777364
		2q13	2:114171139-114321953
		9p24.3-p24.1	9:51374-6659223
		10q11.23-q21.1	10:51917603-54540082
		10q23.2-q26.11	10:89234113-121219507
**region**	**loss**	**homologue region in human**	
		cytoband	position (GRCh37/hg19)
2qA1-qter	1x	10p15.1-p12.1	10:5915452-27157072
		10p12.1	10:27398972-7531240
		2q22.1	2:138721435-139545160
		2q13	2:113723845-114137444
		9q34.11-q34.3	9:131071714-141019156
		9q33.2-q34.11	9:123526077-131061546
		2q22.1-q32.1	2:140065297-188395329
		11q12.1	11:56082416-57753858
		11q11	11:55080583-55323018
		11p11.12	11:51377850-51539057

		11p14.2-p11.2	11:26296397-48658712
		15q13.3-q21.2	15:32906987-51298173
		2q11.1-q11.2	2:95642277-97040617
		2q13	2:111483204-112960231
		2p11.2	2:87345633-87996071
		2q13	2:112973390-113650007
		20p13-p11.21	20:1736101-25606620
		20p13	20:102147-1447942
		20q11.21-q13.32	20:29933153-58056214
		20q13.32-q13.33	20:58148222-62907435
4qC4 –q E2	2x	1p32.1-ptr	1:933238-58547094
		1p31.3-p32.1	1:58654679-67096416
		9p21.2-p24-1	9:6847129-27300708
6qD1 – qE	2x	3q21.2-q21.3	3:125725101-129038484
		3p25.2-p25.1	3:12939278-15163105
		3p14.1-p12.3	3:64017713-75322601
		3p26.3-p25.2	3:61304-12897767
7qA1-qter	1x	19q13.42-q13.43	19:54368915-57485284
		19q13.43	19:58523795-59089552
		19q13.31-q13.33	19:45010010-48707700
		19q12-q13.31	19:30093064-44860951
		19q12	19:28589680-30085362
		19q13.33-q13.41	19:48800017-51921957
		16p13.11	16:16252815-16388674
		11p15.1-p14.3	11:17403485-25251145
		15q11.2	15:22833222-23086601
		15q11.2-q13.1	15:23914751-28586067
		15q13.1-q13.3	15:29107424-32578594
		15q26.3	15:99080385-102265870
		15q26.1-q26.3	15:91593058-99078056
		15q25.3-q26.1	15:85829657-91565912
		15q25.1-q25.3	15:80253398-85682414
		11p11.12	11:49250334-49827246
		11q13.4-q14.3	11:71627032-89350901
		10p11.21	10:37191655-37402201
		11p15.4-p15.1	11:3631069-17360027
		16p13.11	16:15260325-15369270
		16p13.11-p12.3	16:16681590-18325190
		16p12.3-p12.2	16:18608156-21351663
		16p12.2-p11.2	16:21572755-28339524
		16p11.2	16:28390845-29030948
		16p11.2	16:29661006-31520748
		10q26.11-q26.3	10:121224592-135295738
		11p15.5-p15.4	11:192898-3098752
		11q13.3-q13.4	11:68728143-71212974
9qA1-qter	1x	11q14.3-q22.3	11:89860533-107436639
		19p13.2	19:8919008-11689880
		7p14.3-p14.2	7:33134362-36494039
		11q22.3-q25	11:107452617-134843539
		15q21.2	15:51349622-51942502
		15q21.2-q25.1	15:51961808-78956872
		6p12.2-p12.1	6:52656530-55784577

region	breakpoint	homologue region in human	
		cytoband	potential tumor associated genes
		6q13-q14.3	6:74104388-86360515
		15q25.1	15:79042978-80196839
		3q22.3-q24	3:138372654-148087492
		3q22.1-q22.3	3:129931635-138353358
		3p21.31-p21.1	3:46446256-52346387
		3p24.1-p22.2	3:27753690-37261140
		3p22.2-p21.31	3:37269243-46423369
12qA1-qter	1x	2p25.1-p23.3	2:10303009-26361943
		2p25.1	2:9354723-9994801
		2p25.1	2:9996101-10284917
		2p25.3-p25.1	2:140908-9278318
		7q22.3-q31.1	7:105210238-107772185
		7p21.3-p21.1	7:12561752-19748810
		7q31.1	7:107772206-112136146
		14q12-q22.1	14:25157192-52251174
		14q23.1-q32.33	14:58666612-106375879
		7q36.3	7:157225645-158937901
		7p21.1-p15.3	7:19761201-22528893
12qA1	1x (in 16.6%)	2p23.3-p25.1	2:10162883-26139074
14qD3 - qtr	x1	13q14.11-q14.2	13:41469941-49799059
		13q14.3-q33.1	13:53226033-103089581
18qA1-qter	1x	10p11.21	10:35284099-35521818
		10p12.1-p11.22	10:28950711-32678701
		10p12.1	10:27747786-28722506
		10p11.21	10:35676708-37094546
		18p11.32	18:112543-599224
		18q11.1-q12.3	18:18528605-41073893
		2q14.3	2:127805408-128786667
		5q22.1-q22.2	5:110280120-112296881
		5q31.2-q32	5:137225085-147624774
		5q22.2-q23.3	5:112310736-130339352
		5q32-q33.1	5:147647374-150177176
		18p11.22-p11.21	18:10202644-11518916
		18q21.31-q21.32	18:54267924-58201586
		18p11.21	18:11649353-13871680
		18q12.3-q21.31	18:41355914-54244819
		18q22.1-q23	18:66339761-78010601
XqA1-qter	1x	Xp11.23-p11.22	X:48262014-51358982
		Xp21.1-p11.23	X:37364439-47520178
		Xq23-q24	X:115210308-117585111
		Xq24-q27.1	X:117586665-140073167
		Xq27.2-q28	X:140429142-154494231
		Xq23	X:114569624-114885545
		Xp22.31-p22.2	X:8784578-9687806
		Xp22.11-p21.1	X:23850309-37316857
		Xq11.1-q23	X:62853720-114517895
		Yp11.2	Y:4132374-5642381
		Xp11.22-p11.21	X:52987493-56318562
		Xp22.2	X:9688235-9917528
		Xp22.2-p22.11	X:10415591-23849592

4qC4	t	9p21.3	9:21967751-21995300
5qF	t	12q24.31	12:121866902-122018920
6qB3	del	3q21.3	3:126423063-126679249
6qE	inv	3p26.1	3:6811688-7783215
6qG3	inv	12q12.1	12:25562241-25801513
6qD2	inv	3q14.1	3:66429221-66551687
7qE1	t	11p15.4	11:5684425-5959849
10qC1	t	19p13.3	19:361750-376013
11qA1	dic/ t	22q122	22:31884674-32014572
11qB3	dic/ t	17p12	17:11144580-11462196
12qE	inv	14q32.12	14:92788925-92962596
12qB	inv	7q31.1	7:110303110-111202573
13qB	del	5q13.2	5:136310987-136934068
17qA3	t	16q13.3	16:731671-734529
18A1	idic/tric	10p11.21	10:34995171-35221367

BC cell line EMT6/P

region	gain	homologue region in human	
		cytoband	position (GRCh37/hg19)
3 qA1- qF2	1x	8q21.11-q21.3	8:76197937-87035414
		8q12.3-q13.1	8:64075897-67315825
		3q24-q25.1	3:148467535-148965303
		3q26.2-q26.32	3:167857105-178105807
		3q26.32-q27.1	3:178137517-182818465
		4q27-q31.1	4:122242382-141190230
		9p11.2	9:45446787-46098069
		21p11.2	21:10369840-10592667
		9p12	9:42028291-42246766
		9q13	9:68139917-68294733
		13q13.2-q14.11	13:34463185-41254213
		3q25.1-q26.2	3:149055816-167822106
		4q31.23-q32.2	4:150966383-163096512
		1q21.1-q23.1	1:144676687-158154741
		1p22.1-p12	1:93905157-120696915
3 qF2 – qH1	3x	4q26	4:119596924-120703320
		4q22.3-q26	4:95284699-119338945
4 qC5 – qD2	1x	9p24.1-p21.2	9:6847129-27220407
		1p32.1-p31.3	1:59120351-67562260
		1p36.33-p32.2~1	1:894315-59012766
5qB1-qC3	1x	2p23.3	2:26394466-27256616
		2p23.3	2:27256674-27749458
		2p23.3-p23.2	2:27759882-29024462
		18p11.32	18:683166-844532
		22q12.2-q12.3	22:32022117-32511666
		4p16.3	4:1109142-3830658
		4p16.3-p11	4:4184743-49083612
6qA1 – qtr	1x	7q21.2-q21.3	7:92745197-97502117
		7p22.1-p21.3	7:7132996-12536829
		7q31.1-q36.1	7:112138919-149583263
		7q36.1	7:150032467-150558657
		7p15.3-p14.3	7:23254035-33103246
		4q22.1-q22.3	4:89178698-95273100

		4q27	4:121018693-122194687
		1p31.3	1:67631910-68317098
		2p11.2	2:88302422-89174373
		2p13.3-p11.2	2:68715037-87095119
		3q21.2-q21.3	3:125725101-129038484
		3p25.2-p25.1	3:12939278-15163105
		3p14.1-p12.3	3:64017713-75322601
		3p26.3-p25.2	3:61304-12897767
		3q21.3-q22.1	3:129094932-129632650
		10q11.21-q11.22	10:43277986-46218167
		12p13.33	12:66113-2823666
		22q11.1-q11.21	22:17565811-18659740
		12p13.31	12:8071763-9214464
		12p13.33-p13.31	12:2903120-7695890
		12p11.21	12:30985917-31165338
		12p13.31-p11.21	12:9901365-30943693
		12p11.21	12:31424829-32537434
8qA1.1 – qE2	1x	19p13.2	19:7112183-8071013
		13q33.1-q34	13:103533915-115092930
		8p23.3-p23.2	8:591286-5358752
		8p23.2-p23.1	8:5368147-6693649
		13q14.3	13:52435459-53211718
		8p11.23-p11.21	8:36716542-42505949
		8p11.21	8:42691750-43058925
		8p12	8:29190466-36677574
		8p23.1	8:8108776-9640417
		8p23.1-p22	8:12579073-17958954
		4q32.2-q35.2	4:163504024-190884657
		8p22-p21.3	8:18227877-20177976
		19p13.12-p13.11	19:16163040-19774937
		22q12.3	22:33658332-35953121
		4q31.1-q31.23	4:141251922-150892329
		19p13.2-p13.12	19:12745060-14683008
		16q11.2-q22.1	16:46693273-69976105
		16q22.1-q24.3	16:70109527-90110030
		1q42.13-q42.3	1:229404294-235324774
		10p11.22-p11.21	10:33112469-35152269
8qA2 (Mar.)	1x	8p11.23-p11.21	8:36716542-42505949
13 qA2 – qC3	1x	7p14.2-p13	7:36524506-43605930
		6p22.3-p22.1	6:20065223-28502803
		6p25.3-p23	6:181261-15099150
		6p23-p22.3	6:15104709-20060798
		9q22.1-q22.32	9:91031851-97067712
		5q35.2-q35.3	5:173750964-177039611
		5q31.1-q31.2	5:134073478-137090938
		9q21.32-q21.33	9:86231955-90340399
		9q22.32-q22.33	9:97320957-99417669
		9p13.1	9:38810965-40707569
		9q12-q13	9:65585614-65901647
		9p11.2	9:43623473-43941731
		8q22.1	8:97247028-97373828
		5p15.33-p15.31	5:191425-7935441

		5q14.3-q15	5:84566270-96144383
15qA1 - qA2	1x	5p15.31-p12	5:8927745-42888975
15 qA2 - qC	2x	8q22.1-q24.3	8:97446632-146158346
15 qD - qtr	1x	22q12.3-q13.33<br>12p11.1<br>12q12-q13.2	22:35962951-51222438<br>12:33476533-34210697<br>12:38607141-55072925
17qA1 - qE5	1x<br>(in 36%)	6q25.2-q25.3<br>6q27<br>6q25.3-q27<br>6q27<br>5q15-q21.1<br>16p13.3<br>5q35.1<br>6p21.32-p21.2<br>21q22.3<br>19p13.12<br>19p13.2<br>6p22.1-p21.32<br>6p21.2-p12.3<br>3p25.1-p24.3<br>2q12.2-q12.3<br>19p13.3<br>5q21.1-q22.1<br>18p11.32-p11.22<br>2p23.2-p16.3<br>2p16.3-p16.2<br>18p11.32	6:155053083-160101646<br>6:167120855-167552070<br>6:160103032-166797236<br>6:167859539-170893754<br>5:96202316-98405239<br>16:222880-3208490<br>5:171946752-172722349<br>6:33359177-39058058<br>21:43490502-45122943<br>19:15270296-15808207<br>19:8366687-8811037<br>6:29322703-33297218<br>6:39266498-49681826<br>3:16307846-20231899<br>2:107383985-108798215<br>19:4229082-6862967<br>5:102759315-110063021<br>18:2534401-9972541<br>2:29033520-51699597<br>2:51709987-53282184<br>18:861722-2534400
19qA-qtr	1x	11q12.1-q13.3<br>9q21.11-q21.31<br>2q13<br>9p24.3-p24.1<br>10q11.23-q21.1<br>10q23.2-q26.11	11:57844834-68709722<br>9:69086307-82777364<br>2:114171139-114321953<br>9:51374-6659223<br>10:51917603-54540082<br>10:89234113-121219507

**region**	**loss**	**homologue region in human**	
		cytoband	position (GRCh37/hg19)
3qH1 –qH4	1x	1p31.3-p22.2<br>7q11.23	1:68589539-89738135<br>7:76282730-76575579
X qA1 – A6	1x	Xp11.23-p11.22<br>Xp21.1-p11.23<br>Xq23-q24<br>Xq24-q27.1	X:48262014-51358982<br>X:37364439-47520178<br>X:115210308-117585111<br>X:117586665-140073167

**region**	**breakpoint**	**homologue region in human**	
		cytoband	potential tumor associated genes
2qC3	t	2q31.3	2:180809603-180871840
3qF2	inv	1p13.2	1:115590632-115632121
3qH1	dup	4q22.3	4:95679119-96079599
4qC5	inv/ dup	9p21.3	9:23690102-23826335
4qD1	dup	1p33	1:48688357-48714316
5qC3	t,dup	4p12	4:45535770-46535769
5qB1	t	7q36.2	7:153387098-154387097
10qA1	idic	6q25.2	6:152442819-152958936

8qA1	idic	19p13.2	19:6612256-7612255
11qD	t	17q21.2	17:38922490-38928414
12qA1	idic	2p23.3	2:24272571-24286551
13A2	dup	7p14.1	7:37945543-38065297
13qC1	dup	5q14.3	5:87485450-87565293
14qA1	idic	No translation	
15qA2	dup	5p14.2	5:24487209-24645087
15qC	dup	8q23.3	8:116142131-117142130
15qD	dup	8q24.22	8:131792547-132054672
XqF5	t	Xp22.2	X:10413350-10851773
**BC cell line TA3 Hauschka**			
**region**	**gain**	**homologue region in human**	
		cytoband	position (GRCh37/hg19)
3 qA1- qF2	1x	8q21.11-q21.3	8:76197937-87035414
		8q12.3-q13.1	8:64075897-67315825
		3q24-q25.1	3:148467535-148965303
		3q26.2-q26.32	3:167857105-178105807
		3q26.32-q27.1	3:178137517-182818465
		4q27-q31.1	4:122242382-141190230
		9p11.2	9:45446787-46098069
		21p11.2	21:10369840-10592667
		9p12	9:42028291-42246766
		9q13	9:68139917-68294733
		13q13.2-q14.11	13:34463185-41254213
		3q25.1-q26.2	3:149055816-167822106
		4q31.23-q32.2	4:150966383-163096512
		1q21.1-q23.1	1:144676687-158154741
		1p22.1-p12	1:93905157-120696915
3 qF2 – qH1	3x	4q26	4:119596924-120703320
		4q22.3-q26	4:95284699-119338945
4 qC5 – qD2	1x	9p24.1-p21.2	9:6847129-27220407
		1p32.1-p31.3	1:59120351-67562260
		1p36.33-p32.2~1	1:894315-59012766
5qB1-qC3	1x	2p23.3	2:26394466-27256616
		2p23.3	2:27256674-27749458
		2p23.3-p23.2	2:27759882-29024462
		18p11.32	18:683166-844532
		22q12.2-q12.3	22:32022117-32511666
		4p16.3	4:1109142-3830658
		4p16.3-p11	4:4184743-49083612
6qA1 – qtr	1x	7q21.2-q21.3	7:92745197-97502117
		7p22.1-p21.3	7:7132996-12536829
		7q31.1-q36.1	7:112138919-149583263
		7q36.1	7:150032467-150558657
		7p15.3-p14.3	7:23254035-33103246
		4q22.1-q22.3	4:89178698-95273100
		4q27	4:121018693-122194687
		1p31.3	1:67631910-68317098
		2p11.2	2:88302422-89174373
		2p13.3-p11.2	2:68715037-87095119
		3q21.2-q21.3	3:125725101-129038484

		3p25.2-p25.1	3:12939278-15163105
		3p14.1-p12.3	3:64017713-75322601
		3p26.3-p25.2	3:61304-12897767
		3q21.3-q22.1	3:129094932-129632650
		10q11.21-q11.22	10:43277986-46218167
		12p13.33	12:66113-2823666
		22q11.1-q11.21	22:17565811-18659740
		12p13.31	12:8071763-9214464
		12p13.33-p13.31	12:2903120-7695890
		12p11.21	12:30985917-31165338
		12p13.31-p11.21	12:9901365-30943693
		12p11.21	12:31424829-32537434
8qA1.1 – qE2	1x	19p13.2	19:7112183-8071013
		13q33.1-q34	13:103533915-115092930
		8p23.3-p23.2	8:591286-5358752
		8p23.2-p23.1	8:5368147-6693649
		13q14.3	13:52435459-53211718
		8p11.23-p11.21	8:36716542-42505949
		8p11.21	8:42691750-43058925
		8p12	8:29190466-36677574
		8p23.1	8:8108776-9640417
		8p23.1-p22	8:12579073-17958954
		4q32.2-q35.2	4:163504024-190884657
		8p22-p21.3	8:18227877-20177976
		19p13.12-p13.11	19:16163040-19774937
		22q12.3	22:33658332-35953121
		4q31.1-q31.23	4:141251922-150892329
		19p13.2-p13.12	19:12745060-14683008
		16q11.2-q22.1	16:46693273-69976105
		16q22.1-q24.3	16:70109527-90110030
		1q42.13-q42.3	1:229404294-235324774
		10p11.22-p11.21	10:33112469-35152269
8qA2 (Mar.)	1x	8p11.23-p11.21	8:36716542-42505949
13 qA2 – qC3	1x	7p14.2-p13	7:36524506-43605930
		6p22.3-p22.1	6:20065223-28502803
		6p25.3-p23	6:181261-15099150
		6p23-p22.3	6:15104709-20060798
		9q22.1-q22.32	9:91031851-97067712
		5q35.2-q35.3	5:173750964-177039611
		5q31.1-q31.2	5:134073478-137090938
		9q21.32-q21.33	9:86231955-90340399
		9q22.32-q22.33	9:97320957-99417669
		9p13.1	9:38810965-40707569
		9q12-q13	9:65585614-65901647
		9p11.2	9:43623473-43941731
		8q22.1	8:97247028-97373828
		5p15.33-p15.31	5:191425-7935441
		5q14.3-q15	5:84566270-96144383
15qA1 - qA2	1x	5p15.31-p12	5:8927745-42888975
15 qA2 - qC	2x	8q22.1-q24.3	8:97446632-146158346
15 qD - qtr	1x	22q12.3-q13.33	22:35962951-51222438
		12p11.1	12:33476533-34210697

		12q12-q13.2	12:38607141-55072925
17qA1 - qE5	1x (in 36%)	6q25.2-q25.3	6:155053083-160101646
		6q27	6:167120855-167552070
		6q25.3-q27	6:160103032-166797236
		6q27	6:167859539-170893754
		5q15-q21.1	5:96202316-98405239
		16p13.3	16:222880-3208490
		5q35.1	5:171946752-172722349
		6p21.32-p21.2	6:33359177-39058058
		21q22.3	21:43490502-45122943
		19p13.12	19:15270296-15808207
		19p13.2	19:8366687-8811037
		6p22.1-p21.32	6:29322703-33297218
		6p21.2-p12.3	6:39266498-49681826
		3p25.1-p24.3	3:16307846-20231899
		2q12.2-q12.3	2:107383985-108798215
		19p13.3	19:4229082-6862967
		5q21.1-q22.1	5:102759315-110063021
		18p11.32-p11.22	18:2534401-9972541
		2p23.2-p16.3	2:29033520-51699597
		2p16.3-p16.2	2:51709987-53282184
		18p11.32	18:861722-2534400
19qA-qtr	1x	11q12.1-q13.3	11:57844834-68709722
		9q21.11-q21.31	9:69086307-82777364
		2q13	2:114171139-114321953
		9p24.3-p24.1	9:51374-6659223
		10q11.23-q21.1	10:51917603-54540082
		10q23.2-q26.11	10:89234113-121219507
**region**	**loss**	**homologue region in human**	
		cytoband	position (GRCh37/hg19)
3qH1 –qH4	1x	1p31.3-p22.2	1:68589539-89738135
		7q11.23	7:76282730-76575579
X qA1 – A6	1x	Xp11.23-p11.22	X:48262014-51358982
		Xp21.1-p11.23	X:37364439-47520178
		Xq23-q24	X:115210308-117585111
		Xq24-q27.1	X:117586665-140073167
**region**	**breakpoint**	**homologue region in human**	
		cytoband	potential tumor associated genes
2qC3	t	2q31.3	2:180809603-180871840
3qF2	inv	1p13.2	1:115590632-115632121
3qH1	dup	4q22.3	4:95679119-96079599
4qC5	inv/ dup	9p21.3	9:23690102-23826335
4qD1	dup	1p33	1:48688357-48714316
5qC3	t,dup	4p12	4:45535770-46535769
5qB1	t	7q36.2	7:153387098-154387097
10qA1	idic	6q25.2	6:152442819-152958936
8qA1	idic	19p13.2	19:6612256-7612255
11qD	t	17q21.2	17:38922490-38928414
12qA1	idic	2p23.3	2:24272571-24286551
13A2	dup	7p14.1	7:37945543-38065297
13qC1	dup	5q14.3	5:87485450-87565293

14qA1	idic	No translation	
15qA2	dup	5p14.2	5:24487209-24645087
15qC	dup	8q23.3	8:116142131-117142130
15qD	dup	8q24.22	8:131792547-132054672
XqF5	t	Xp22.2	X:10413350-10851773
